# A new bush anole (Iguanidae, Polychrotinae, *Polychrus*) from the upper Marañon basin, Peru, with a redescription of *Polychrus peruvianus* (Noble, 1924) and additional information on *Polychrus gutturosus* Berthold, 1845

**DOI:** 10.3897/zookeys.141.1678

**Published:** 2011-10-28

**Authors:** Claudia Koch, Pablo J. Venegas, Antonio Garcia-Bravo, Wolfgang Böhme

**Affiliations:** 1 Zoologisches Forschungsmuseum Alexander Koenig, Adenaueralle 160, 53113 Bonn, Germany; 2 Centro de Ornitología y Biodiversidad (CORBIDI), Santa Rita 117, Huertos de San Antonio, Surco, Lima, Perú

**Keywords:** Andes, dryforest, new species, lizard, bush anoles, reptiles, *Polychrus jacquelinae* sp. n., *Polychrus peruvianus*, *Polychrus gutturosus*, *Polychrus spurrelli*

## Abstract

We herein describe a new colorful species of *Polychrus* with a conspicuous sexual dimorphism from the dry forest of the northern portion of Región de La Libertad, Peru. The new species differs from all other *Polychrus* species, in that this species has very small dorsal scales and thus a higher number of scales around midbody and in the middorsal line from behind the occipital scales to the level of the posterior edge of the thigh. Furthermore, we redescribe *Polychrus peruvianus* whose original description is short and lacks information on intraspecific variation and sexual dimorphism. Also, we add some information on intraspecific variation and ecology of *Polychrus gutturosus*. Finally, we synonymize *Polychrus spurrelli* Boulenger with *Polychrus gutturosus*.

## Abstract (es)

**Resúmen**

Describimos una colorida nueva especie de *Polychrus*, caracterizada por un acentuado dimorfismo sexual, proveniente de los bosques secos situados en la porción noreste de la Región de La Libertad en Perú. Esta nueva especie se diferencia de las otras especies de *Polychrus* por poseer escamas dorsales muy pequeñas que se presentan en mayor cantidad, alrededor de la zona media del cuerpo y a lo largo de la línea media dorsal, desde detrás de las escamas occipitales hasta el borde posterior de los muslos. Además, redescribimos la especie *Polychrus peruvianus*, debido a su breve descripción original que no incluye información sobre la variación intraespecífica ni el dimorfismo sexual. También agregamos información sobre la variación intraespecífica y ecología de *Polychrus gutturosus*. Finalmente, optamos por sinonimizar *Polychrus spurrelli* Boulenger con *Polychrus gutturosus*.

## Introduction

The polychrotine iguanid lizards of the genus *Polychrus* (Cuvier, 1817) occur in Central America northward to Nicaragua and in large parts of South America, on both sides of the Andes (Avila-Pires 1995). The genus is composed of six species: *Polychrus acutirostris*Spix, 1825; *Polychrus femoralis* Werner, 1910; *Polychrus gutturosus* Berthold, 1845; *Polychrus liogaster* Boulenger, 1908; *Polychrus marmoratus* (Linnaeus, 1758); *Polychrus peruvianus* (Noble, 1924), of which four are believed to occur in Peru (*Polychrus femoralis*, *Polychrus liogaster*, *Polychrus marmoratus*, and *Polychrus peruvianus*). Boulenger (1914) described *Polychrus spurrelli* as a seventh species in the genus which is, however, currently considered to be a subspecies of *Polychrus gutturosus* by many herpetologists (e.g. Parker 1935, Peters 1967, Peters and Donoso-Barros 1970, 1986). However, the status of this taxon is still unclear.

Although the existence of all the currently recognized species in the genus has been known for quite a long time, with the latest discovered species being described more than 86 years ago (Noble 1924), little is still known about most of the species. Original descriptions of all species in this genus are brief and lack information on intraspecific variation and sexual dimorphism. Therefore, they seem to be inadequate by today's standards.

Avila-Pires (1995) gave detailed redescriptions of *Polychrus acutirostris*, *Polychrus liogaster* and *Polychrus marmoratus*. Savage (2002) gave a more detailed redescription of an unknown number of male and female specimens of *Polychrus gutturosus* but, as did the original description (Berthold 1845), he failed to provide scale counts. Taylor (1956) provided a detailed description with some measurements and scale counts on two individuals but the small number of specimens still tells little regarding variation. We examined 27 specimens from museum collections and will herein provide more information on intraspecific variation based on measurements and scale counts.

We further redescribe *Polychrus peruvianus*, a comparatively common species which occurs in northern Peru and southern Ecuador. Originally, Noble (1924) described this species as belonging to a new genus *Polychroides*, and thus did not compare it with other species of the genus *Polychrus*, where it was later placed by Etheridge (1965). The original description is based on only a single male individual and thus information on intraspecific variation and on female specimens is still lacking. Yánez-Muñoz et al. (2006) and Schlüter (2010) recently provided a brief diagnoses for this species but failed to provide a comprehensive description.

To contribute to the knowledge of the herpetofauna of Andean dry forests, three of the authors (P. J. Venegas, A. W. Garcia Bravo, and C. Koch) surveyed the inter-Andean valleys of the upper Marañon basin between March 2008 and November 2010. The investigations resulted in the discovery of an obviously undescribed species of *Polychrus* which is described herein. During their fieldwork, they also collected 47 *Polychrus peruvianus* and were able to note several important data on the natural history of this species.

## Materials and methods

All collected specimens were preserved in 96% ethanol and stored in 70% ethanol. The new species is described on the basis of 6 collected specimens (2 males and 4 females). The redescription of *Polychrus peruvianus* is based on 47 specimens (24 males, 23 females), and the information given on *Polychrus gutturosus* is based on 27 specimens (10 males, 15 females, 2 undefined juvenile) including the holotype. We further examined the two female syntypes of *Polychrus spurrelli* and two male specimens that were deposited under this species epithet in the British Museum of Natural History, London, England.

Comparative data for other *Polychrus* species were taken from Avila-Pires (1995), in addition to the examination of preserved specimens housed in the Museo de Historia Natural San Marcos, Lima, Peru (MUSM), the Centro de Ornitología y Biodiversidad, Lima, Peru (CORBIDI), the Senckenberg Forschungsinstitut und Naturmuseum, Frankfurt, Germany (SMF), the Museo de Zoología de la Pontificia Universidad Católica del Ecuador, Quito, Ecuador (QCAZ), the División de Herpetología, Museo Ecuatoriano de Ciencias Naturales, Quito, Ecuador (DHMECN), the British Museum of Natural History, London, England (BM), the Muséum d'Histoire Naturelle, Geneva, Switzerland (MHNG), and the Zoologisches Forschungsmuseum Alexander Koenig, Bonn, Germany (ZFMK). All measurements of the head, body, and limbs were taken with a Vernier caliper (to the nearest 0.1 mm), tail measurements were taken with a tape measure. To facilitate comparisonwithin the genus we tried to structure our species descriptions analog to that of Avila-Pires (1995) and used the same terminology in the diagnoses and descriptions. In the tables, ranges of morphometric and pholidosis characters are presented, followed by mean values and standard deviations in parenthesis.

Altitudes above sea level and geographic coordinates were determined with a GPS (Garmin GPSMap 60CSx) using the geodetic datum WGS84. Humidity and air temperatures were taken with a digital thermo-hygrometer (Extech) with an external sensor.

## Results

### Polychrus jacquelinae sp. n.

urn:lsid:zoobank.org:act:A5E0F6FD-F3A5-4DDE-9827-9D3D485E4682

http://species-id.net/wiki/polychrus_jacquelinae

#### Holotype.

**Figure 1.**
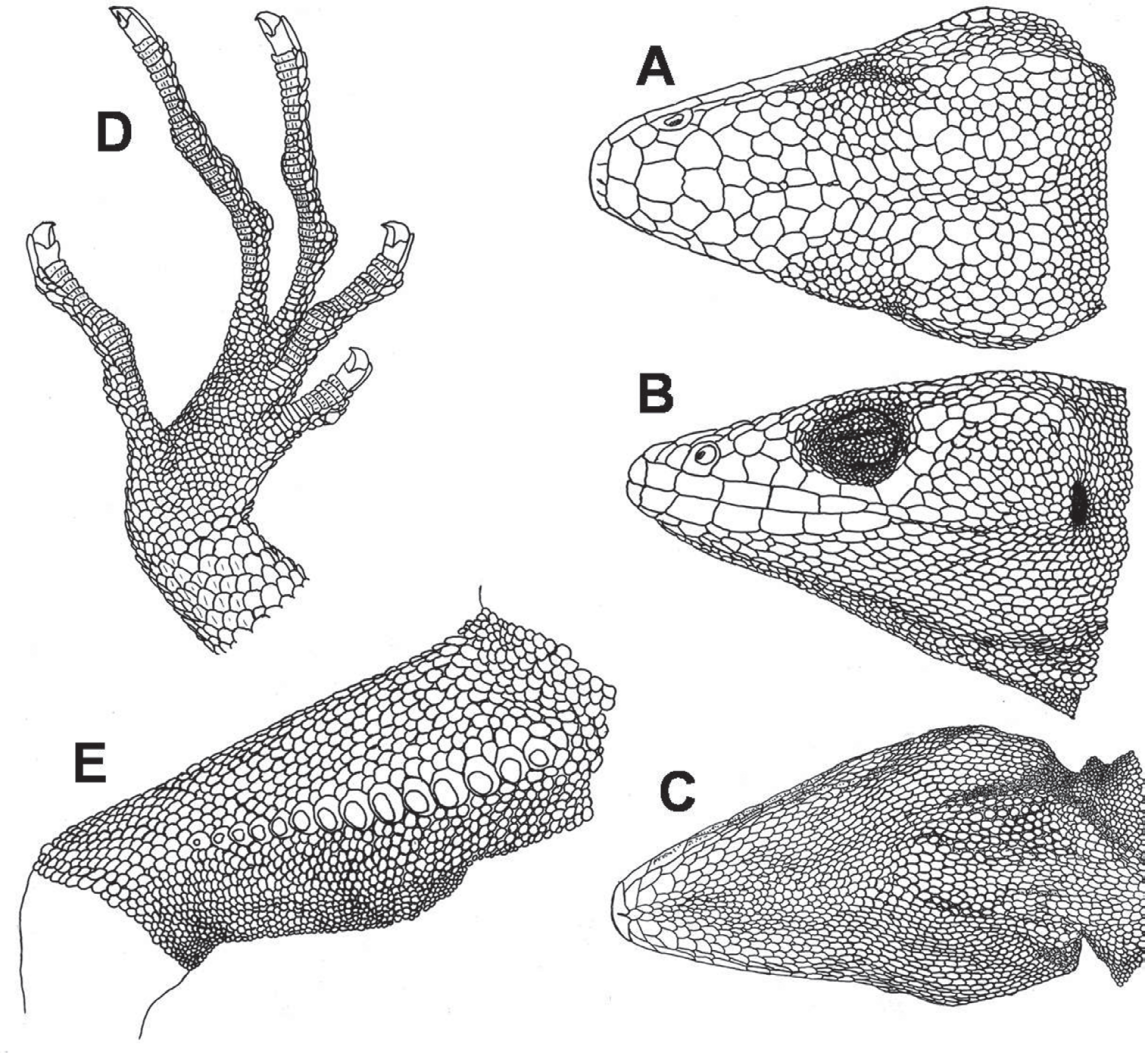
Male holotype of *Polychrus jacquelinae*sp. n. CORBIDI 7725 dorsal **A**, lateral **B** and ventral **C** views of head, ventral aspect of right foot **D**, ventral view of right thigh with femoral pores **E**.

**Figure 2.**
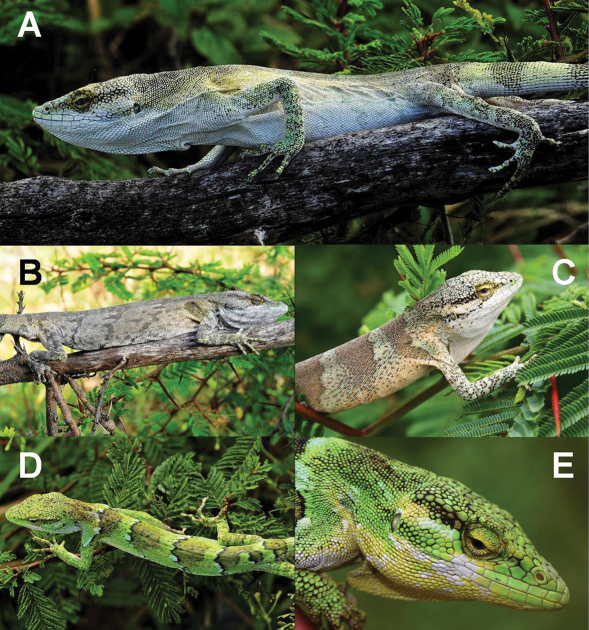
*Polychrus jaqcuelinae* sp. n. from La Libertad, Peru: male holotype CORBIDI 7725 with normal colouration **A** photograph by M. León, in stress colouration **B** photograph by M. León, subadult male **C** ZFMK 91763, female **D** CORBIDI 5742, photograph by M. León, close-up of the head of one female **E**, ZFMK 91764.

CORBIDI 7725 (Fig. 1A–E, 2A, B), an adult male from a new road, that was still under construction and is intended to connect San Vicente/Pusaq and Uchumarca (06°59'S, 77°54'W, approximately 1460–1570m above sea level), Province Bolivar, Región de La Libertad, Peru, collected by W.A. Garcia Bravo on 01 July 2010.

#### Paratypes.

 CORBIDI 5742 (Fig. 2D) and CORBIDI 7724, two adult females collected with the holotype; ZFMK 91763 (Fig. 2C) subadult male and ZFMK 90834, ZFMK 91764 (Fig. 2E) two adult females from the type locality, collected by W.A. Garcia Bravo and C. Koch on 24 April 2009.

#### Diagnosis.

 (Tab. 1). (1) A *Polychrus* with a maximum known snout-vent-length (SVL) of 144 mm; (2) dorsal and gular crests absent; (3) 131 to 186 scales around midbody; (4) 198 to 215 scales in middorsal row from behind the occipital scales to the level of the posterior edge of the thigh; (5) femoral pores 13 to 15 on one side; (6) lamellae on finger IV 33–36; (7) lamellae on toe IV 42–48; (8) tail 2.13–2.33 times SVL; (9) dorsal and ventral scales small and smooth (10) a prominent sexual dichromatism present.

**Table 1.** Summary of morphometric and pholidosis characters of *Polychrus jacquelinae* sp. n.

Sex	All (n=6)	Males (n=2)	Females (n=4)
Axilla-groin length/SVL	0.46–0.55<br/> (0.48 ± 0.03)	0.46–0.5<br/> (0.48 ± 0.03)	0.48–0.55<br/> (0.51 ± 0.03)
Head length/SVL	0.23–0.25<br/> (0.24 ± 0.01)	0.25<br/> (0.25 ± 0.00)	0.23–0.24<br/> (0.24 ± 0.01)
Head length/Head width	1.46–1.63<br/> (1.55 ± 0.06)	1.51–1.56<br/> (1.54 ± 0.04)	1.46–1.63<br/> (1.56 ± 0.08)
Head width/Head height	0.98–1.1<br/> (1.03 ± 0.05)	1.01–1.06<br/> (1.04 ± 0.03)	0.98–1.1<br/> (1.03 ± 0.06)
Tail length/SVL	2.13–2.33<br/> (2.23 ± 0.08)	2.16–2.22<br/> (2.19 ± 0.04)	2.13–2.33<br/> (2.25 ± 0.09)
Scales around midbody	131–186<br/> (164.17 ± 20.45)	139–186<br/> (162.5 ± 33.23)	131–149<br/> (138 ± 7.87)
Vertebral scales	198–215<br/> (206.17 ± 6.94)	198–202<br/> (200 ± 2.83)	202–215<br/> (209.25 ± 6.29)
Gular scales	72–83<br/> (75.67 ± 4.18)	73–83<br/> (78 ± 7.07)	72–78<br/> (74.5 ± 2.65)
Diameter eye/head length	0.17–0.23<br/> (0.19 ± 0.02)	0.18–0.19<br/> (0.18 ± 0.01)	0.17–0.23<br/> (0.19 ± 0.03)
Subdigitals finger IV	33–36<br/> (34.67 ± 1.21)	34–35<br/> (34.5 ± 0.71)	33–36<br/> (34.75 ± 1.5)
Subdigitals toe IV	42–48<br/> (45.33 ± 2.16)	46–47<br/> (46.5 ± 0.71)	42–48<br/> (44.75 ± 2.5)
Forelimbs/SVL	0.38–0.46<br/> (0.42 ± 0.07)	0.41–0.42<br/> (0.42 ± 0.01)	0.38–0.46<br/> (0.42 ± 0.03)
Hindlimbs/SVL	0.51– 0.59<br/> (0.53 ± 0.07)	0.51–0.53<br/> (0.52 ± 0.01)	0.52–0.59<br/> (0.54 ± 0.04)
Femoral pores (left)	13–15<br/> (14 ± 0.63)	14<br/> (14 ± 0)	13–15<br/> (14 ± 0.82)

*Polychrus jacquelinae* sp. n. differs from other species of *Polychrus* by having more than 130 scales around midbody and more than 198 scales in middorsal row from behind the occipital scales to the level of the posterior edge of the thigh (*Polychrus acutirostris* has fewer than 73 and 126 scales; *Polychrus femoralis* fewer than 100 and 156; *Polychrus gutturosus* fewer than 81 and 105; *Polychrus liogaster* fewer than 95 and 125; *Polychrus marmoratus* fewer than 90 and 118; and *Polychrus peruvianus* fewer than 74 and 70 (paravertebrals), respectively). Furthermore, the new species is easily distinguished from *Polychrus acutirostris* and *Polychrus femoralis* by the absence of keeled ventral scales; from *Polychrus gutturosus* by the absence of multicarinate ventral scales and by the presence of very small gular scales; from *Polychrus liogaster* and *Polychrus marmoratus* by the presence of a sexual dimorphism in colouration (absent in the two latter species); and from *Polychrus peruvianus* by the absence of vertebral and gular crests.

#### Description of holotype.

 Adult male with a snout-vent-length (SVL) of 140.5 mm. Head 0.25 times SVL, 1.51 times longer than wide, as wide as high. Snout pointed; canthus rostralis distinct posteriorly. Neck narrower than the head, and almost as wide as the anterior part of the body. Body compressed. Limbs well developed, forelimbs 0.41 times SVL, hindlimbs 0.53 times SVL, tibia 0.17 times SVL. The tail almost round in cross section, tapering toward the tip; 2.22 times SVL. Rostral trapezoid, almost two times as wide as high, visible from above. Posterior margin with 3 sutures that do not partition the rostral, bordered posteriorly by 2 large scales. Scales on snout heterogeneous in size, irregularly polygonal, juxtaposed, flat, rugose, some are swollen; 3 scales across snout between second canthals. 3 canthals between nasal and supraciliaries, anterior one wide. Supraorbital semicircles more or less distinct, with 9–10 scales, separated medially by 1 row of scales, slightly smaller in size than those of supraorbital semicircles (Fig. 1A). Scales on supraocular region distinctly smaller than those on snout, polygonal to rounded, juxtaposed, flat, and smooth, irregularly arranged, except for a row of smaller scales adjacent to supraciliaries. Supraciliaries 12–13, juxtaposed, smooth, anterior ones slightly longer; in a continuous series with canthals. 15 supraoculary scales on the dorsal surface of the orbit counted in a transverse line across its greatest width. Scales on parietal region, irregular polygonal, some almost rounded, juxtaposed, flat, smooth, intermediate in size between those on snout and on supraocular region. Scales on interparietal region polygonal, juxtaposed, rugose, some are somewhat swollen. Parietal eye absent. Loreal region with two scales. Nostril directed laterally, in the centre of a single nasal. Nasal in contact with second supralabial. 5 internasals. Eye diameter 0.18 times as long as head length. Eyelids partially fused, covered by granules of almost same size throughout the eyelids. A continuous series of 2 preoculars, 3–4 suboculars, which are in direct contact with supralabials, and 4 postoculars. Supralabials 7; followed to commissure of mouth by 1–2 relatively small scales. Temporal region with polygonal or rounded, juxtaposed, flat, and smooth scales, smaller toward the ear opening; delimited dorsally by a single row of enlarged supratemporal scales. Ear opening vertically oval, with smooth margin; tympanum superficial (Fig. 1B).

Mental bell-shaped, almost 2.5 times as wide as high, posterior half divided by a median sulcus. Postmentals 3, lateral ones distinctly larger than median scale. Infralabials 6; followed to commissure by 2–3 distinctly smaller scales. Lateral scales on chin almost the same size as those on median part of chin, irregular polygonal, juxtaposed, flat, smooth, and slightly convex. No elevated scales on the median part of chin and gular region present. Gular fan has scales the same size and structure as chin scales, which are separated from each other by an extensible skin covered with granules (Fig. 1C). Gular fan reaches level of forelimbs. 73 gular scales in transverse line between the two tympani.

Scales on nape anteriorly relatively small, granular and almost rounded, juxtaposed, convex; posteriorly grading into dorsals. Scales on the sides of the neck are slightly larger than those on the nape, merging ventrally with the gulars. Dorsals polygonal to rounded, juxtaposed, flat, to some extent convex; 198 scales in a middorsal line between the occiput and the posterior margin of the hindlimbs. Scales on flanks are of a similar size and same shape as those of dorsum, convex, smooth, in poorly defined, oblique rows. Ventrals larger than dorsals, flat, smooth, lanceolate, and imbricate, in poorly defined, oblique and transverse rows. A gradual transition between dorsal, lateral and ventral scales. Scales around midbody 186. Preanal plate has scales which are smaller than ventrals, approximately the same size as dorsals. Preanal pores absent. Femoral pores 14 per side, almost in the centre of a slightly larger scale (Fig. 1E).

Tail has rhomboid, flat, sharply keeled scales, distinctly larger than the dorsals, arranged in longitudinal and oblique rows; keels aligned longitudinally; on ventral surface of the tail, scales are slightly larger and more rectangular. Tail not regenerated ending somewhat bluntly.

Scales on forelimbs slightly larger than dorsals, polygonal to rounded, flat, smooth, mostly imbricate but more juxtaposed on upper arm; slightly smaller on ventral aspect of forearms; towards posterior aspect ventrals become smaller. Scales on hindlimbs are as large as dorsals, polygonal to rounded, flat, smooth, imbricate on thigh and ventrally, juxtaposed on dorsal surface of tibia, slightly larger and slightly keeled on ventral part of tibia, and slightly lanceolate; toward posterior aspect of thighs, both dorsally and ventrally, scales become distinctly smaller. Subdigital lamellae of fingers and toes single, short, multicarinate, 35 under fourth finger, 47 under fourth toe (Fig. 1D).

#### Measurements of holotype (in mm).

 Snout-vent length 140.5; axilla-groin length 64; length of leg 74; length of arm 58; length of tail 311.5; body height at midbody 25.3; body width at midbody 22.4; length of head 35.7; height of head 23.4; width of head 23.7; length of snout 25; diameter of eye 6.3; distance from tip of snout to anterior margin of nostril 7.1; distance from nostril to eye 7.9; distance from eye to ear 14.

#### Colouration of holotype.

 In life, when unstressed (Fig. 2A), the dorsal surface of the head of the male holotype is grey to greenish-grey on the snout and the parietal region, muddy yellow on the frontal region and more or less shiny yellow on the supraocular region. Most of the dorsal head scales as well as the dorsal body scales have dark edges. The sides of the head are white to slightly yellow. The granules on the eyelids and the scales surrounding the eye are shiny yellow. The dorsum is pale grey, and in some parts fades to a pale yellow or pale green with hardly any special markings, except for some slightly darker saddle blotches. The body parts above and behind the insertion of the front legs are almost as shiny yellow as the granules of the eyelids. The dorsal surface of the limbs is pale grey-green and of the tail white-grey. The tail is ringed with 12 darker grey bands, with each band being 10–13 scales in width, and thus of the same width as the ground coloured interspaces. The bands are indistinct at the beginning but become more distinct towards the tip. The chin scales, gular fan, venter and ventral surface of the limbs are white.

Under stress (Fig. 2B), colouration of whole body changes into a moderate grey, being the darkest in the middorsal region. Colouration grades laterally into beige, especially behind the limbs, with darker, frazzled, transverse stripes on the dorsum and limbs.

The species may also change its colours to camouflage itself, but this behaviour was not observed during our short investigation.

In preservative, the general dorsal colour is mainly brown-grey and the head and middorsal stripe are darker than the lateral body parts. Bands on the tail alternate in pale brown-grey and dark grey-brown. The ventral region is white to pale grey.

#### Variation. 

(Tab. 1) Female paratypes (n=4) reach a maximum SVL of 144 mm and a maximum tail length of 306 mm. Tails of paratypes (n=5) are 2.13–2.33 times SVL and axilla-groin lengths are 0.48–0.55 times SVL. Heads reach 0.23–0.25 times SVL, are 1.46–1.63 as long as they are wide and 0.98–1.1 times as wide as they are high. Eye diameter is 0.17–0.23 times the head length. Scales around the midbody vary between 131**–**149 and the vertebral scale number from behind the occiput to the level of the posterior edge of the thigh varies between 202–215. The rostral scale is posteriorly bordered by 2–4 scales and exhibits 1–4 posterior sutures that do not completely divide the rostral. There are three scales across the snout between the second canthals. There are three to four canthals between the nasal and the superciliaries. The supraorbital semicircles consist of 9–10 scales, and are separated medially by one to two rows of scales. Superciliaries 9–13 and supraocularies 13–17. In 4 paratypes, the nasal was in contact with the second supralabial, and in one specimen it was separated from the labials by small scales.

There is a continuous series of 2–4 preoculars, 3 suboculars, which are in direct contact with the supralabials, and 3–4 postoculars. Supralabials 5–7; followed to commissure of mouth by 1–4 scales. Internasals in all paratypes 5. Supratemporals 6–7.

Mental half divided by a median sulcus in three paratypes, almost divided in one paratype and completely divided in another specimen. Postmentals 4 in all paratypes. Gular scales 72–83. Infralabials 6–8; followed to commissure by 2–6 scales. Femoral pores 13–15 per side. Subdigital lamellae 33–36 under fourth finger, 42–48 under fourth toe.

#### Colour variation.

In life, the dorsal surface of the head of the subadult male paratype (ZFMK 91763, Fig. 2C) is pale grey-green on the snout and frontal region, grey-blue in the parietal region and pale green-yellow on the supraocular region. Most dorsal head scales as well as dorsal body scales have dark edges. The sides of the head are pale green-grey to white in the region anterior to the eye, and pale peach-coloured in the temporal region. There is a dark stripe from the posterior margin of the eye, reaching below the supratemporals to the level of the ear opening. The labials and suboculars are mostly white and the dorsum is pale mint green with 6 brown to peach-coloured saddle blotches, 16–29 scales in width, being widest on the middorsal part and tapering towards the flanks. The dorsum is spotted black. There is a mint colour grading into pale peach-colour on the sides of the neck and flanks. The dorsal surface of the forelimbs is mint green and of the hindlimbs, it is peach-coloured intermixed with some mint scales. Both front and hindlimbs are spotted black. The ground colour of the tail is pale beige with 12 darker brownish bands, 10–18 scales in width. The venter, chin scales, gular fan, and limbs are white to whitish-grey.

In life, the dorsal ground colouration of females (CORBIDI 5742, CORBIDI 7724, ZFMK 90834, ZFMK 91764) on the head, back, limbs and tail is lime green with most parts spotted black (Fig. 2D); granules of eyelids are shiny yellow to lime green (Fig. 2E); sides of head posteriorly of eye between supratemporals and the beginning of the gular fan in some of the specimens are intermixed with numerous blue toned scales; dorsum has 5–6 undulated transversal black bands, 2–6 scales in width, first band on level of forelimbs, is continued on the limbs, last one on level of hindlimbs; bulges of the undulated black lines are anteriorly filled with bluish blotches, 3–5 scales in width; posteriorly, the black bands are followed by darkly shaded green stripes, 12–20 scales in width; followed by ground colour, 8–10 scales in width; adjacent starts the repetition of the whole pattern, beginning with the bluish blotches, followed by the undulated transversal black band; tail with 12 darkly shaded greenish bands, 9–12 scales in width and of almost same width as ground colour interspaces. On ventral surface, chin scales and gular fan are shiny yellow, and in some parts, spotted with white (Fig. 2E); one gravid female (ZFMK 91764) has pale green chin scales and pale orange gular fan. Venter and limbs are white; tail white to whitish-grey annulated with darker grey bands, indistinct at the beginning but becoming more distinct towards the tip. No colour changes were observed in the female specimens of this species.

In preservative, dorsal pattern remains similar to the pattern in life but colouration mainly consist of different shades of blue, only the darkly shaded green stripes on the dorsum are brownish-blue to brown in some of the specimens; head grey-blue to greenish-blue; on tail brownish to greyish-brown bands, alternating with pale greyish-brown, pale green or blue toned bands. On ventral surface, gular fan, venter, limbs and tail white to greyish-white; chin also white to greyish white, but in one gravid specimen (ZFMK 91764) it is intermixed with pale bluish scales.

#### Etymology.

 The species is dedicated to Jacqueline Maria Charles (Leicester, England) in recognition of her support of nature conservation and taxonomic research through the BIOPAT initiative.

#### Distribution and natural history.

**Figure 3.**
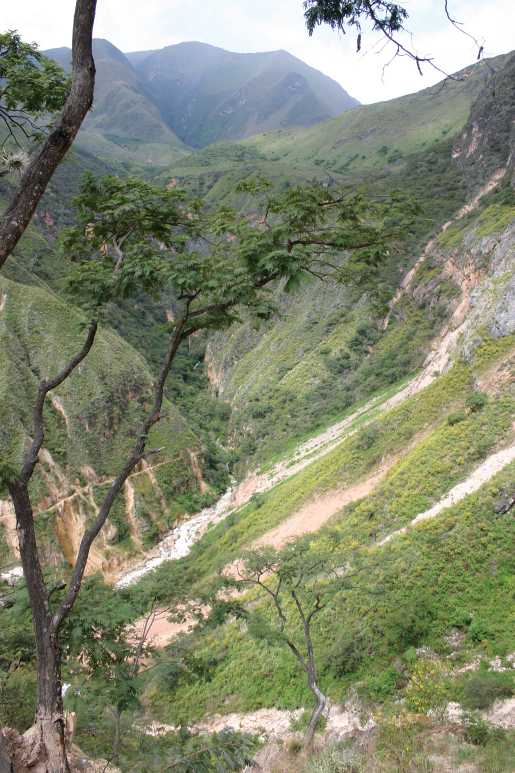
The type locality of *Polychrus jacquelinae*sp. n. CORBIDI 7725 near San Vicente, La Libertad, Peru.

This new species is only known from the type locality (Fig. 3) in the inter-Andean valley on the western slope of the northern portion of the Cordillera Central (see Duellman and Pramuk 1999), at an elevation of 1460 to 1570 m above sea level. All individuals were collected near a road between San Vicente/Pusaq and Uchumarca (06°59'S, 77°54'W), Province Bolivar, Región de La Libertad, Peru. *Polychrus jacquelinae* inhabits the equatorial dry forest eco-region in the upper Marañón basin, fide Brack (1986). One subadult male (ZFMK 91763) and two females (ZFMK 90834, ZFMK 91764) were found on 24 April 2009 between 9.45 p.m. and midnight, sleeping in trees of *Acacia macracantha* in heights between 1.8 m and 5 m above the ground, at an air temperature of 22°C and a humidity of 63%. One adult male (CORBIDI 7725) and two adult females (CORBIDI 5742, CORBIDI 7724) were found on 01 July 2010 between 7.30 p.m. and 8.30 p.m., sleeping, at an air temperature of 25°C and a humidity between 34–38%, in a bush of Fabaceae sp. approximately 2 m above the ground, in a bush of *Croton* sp. approximately 4 m above the ground, and in a tree of *Bombax* sp. approximately 3.5 m above the ground, respectively. One female (ZFMK 91764) was gravid and contained 6 oval eggs (3 in each of the oviducts). On average, these eggs had a length of 27.7 mm and a width of 15.8 mm.

### Polychrus peruvianus

(Noble, 1924)

http://species-id.net/wiki/polychrus_peruvianus

Polychroides peruvianus  Noble, Occasional Papers of the Boston Society of Natural History, 5: 109. – Terra typica: near Querocotilla, province of Cajamarca, Peru. – 1924
Polychroides peruvianus  – Burt and Burt, Transactions of the Academy of Science of St. Louis, 28: 40. – 1933
Polychrus peruvianus  — Etheridge, Herpetologica, 21: 167. – 1965
Polychrus peruvianus  — Gormanet al., Breviora, 316: 5. – 1969
Polychroides peruvianus  – Peters and Donoso-Barros, Smithsonian Institution Press, Washington D.C. & London: 232. – 1970
Polychroides peruvianus  – Peters and Donoso-Barros, Smithsonian Institution Press, Washington D.C. & London: 232. – 1986
Polychrus peruvianus  – Lehr, Natur und Tier-Verlag: 203. – 2002
Polychrus peruvianus  – Yánez-Muñozet al., Check List, 2 (2): 63. – 2006

#### Diagnosis

 (Tab. 2). (1) A *Polychrus* with a maximum known SVL of 152 mm; (2) males larger than females; (3) a prominent dorsal and gular crest present; (4) 52 to 74 scales around midbody; (5) 56 to 70 paravertebral scales from the occipital region to the level of the posterior edge of the thigh; (6) femoral pores 6 to 13 on one side; (7) lamellae on finger IV 25–33; (8) lamellae on toe IV 32–43; (9) tail 1.29–3.15 times longer than SVL; (10) paravertebral scales unicarinate; (11) ventral scales uni- to tricarinate, rarely multicarinate; (12) gular scales oval, striated, much larger than ventrals; (13) a prominent sexual dichromatism present.

**Table 2.** Summary of morphometric and pholidosis characters of *Polychrus peruvianus*

Sex	All (n=47)	Males (n=24)	Females (n=23)
Axilla-groin length/SVL	0.43–0.53<br/> (0.49 ± 0.03)***	0.43–0.53<br/> (0.47 ± 0.03)**	0.47–0.52<br/> (0.5 ± 0.02)*
Head length/SVL	0.21–0.28<br/> (0.25 ± 0.02)	0.24–0.28<br/> (0.26 ± 0.01)	0.21–0.26<br/> (0.24 ± 0.01)
Head length/Head width	1.37–1.84<br/> (1.58 ± 0.10)***	1.37–1.66<br/> (1.54 ± 0.09)**	1.49–1.84<br/> (1.61 ± 0.09)*
Head width/Head height	0.69–1.08<br/> (0.95 ± 0.09)***	0.84–1.06<br/> (0.96 ± 0.06)**	0.69–1.08<br/> (0.93 ± 0.11)*
Tail length/SVL	1.29–3.15<br/> (2.71 ± 0.47)	1.53–3.15<br/> (2.67 ± 0.55)	1.29–3.11<br/> (2.76 ± 0.21)
Scales around midbody	52–74<br/> (61.49 ± 5.15)	52–67<br/> (58.75 ± 3.42)	56–74<br/> (64.35 ± 4.92)
Elevated vertebrals (crest)	9–28<br/> (22.19 ± 6.16)	20–28<br/> (25.92 ± 1.77)	9–28<br/> (18.3 ± 6.82)
Gular scales	28–38<br/> (33.7 ± 2.58)***	28–36<br/> (32 ± 2.36)**	31–38<br/> (35 ± 1.96)*
Diameter eye/head length	0.25–0.31<br/> (0.27 ± 0.02)***	0.25–0.28<br/> (0.26 ± 0.01)**	0.25–0.31<br/> (0.28 ± 0.02)*
Subdigitals finger IV	25–33<br/> (29.74 ± 1.81)	28–33<br/> (30.13 ± 1.54)	25–33<br/> (29.35 ± 2.06)
Subdigitals toe IV	32–43<br/> (37.15 ± 3.01)	33–43<br/> (36.75 ± 3.0)	32–41<br/> (37.57 ± 3.1)
Forelimbs/SVL	0.46–0.57<br/> (0.52 ± 0.03)***	0.46–0.57<br/> (0.53 ± 0.03)**	0.48–0.57<br/> (0.51 ± 0.03)*
Hindlimbs/SVL	0.58–0.69<br/> (0.61 ± 0.04)***	0.58–0.69<br/> (0.63 ± 0.04)**	0.52–0.65<br/> (0.59 ± 0.04)*
Femoral pores (left)	6–13<br/> (9.74 ± 1.45)	6–13<br/> (9.77 ± 1.55)	7–12<br/> (9.15 ± 1.34)

***(n=23), **(n=10), *(n=13)

#### Description.

A *Polychrus* with a maximum SVL in males of 152 mm, in females of 147 mm. Head 0.21–0.28 times SVL, 1.37–1.84 times as long as wide and 0.69–1.08 times as wide as high. Snout bluntly pointed; canthus rostralis well pronounced. Neck narrower than the head, and slightly narrower than the anterior part of the body. Limbs well developed, forelimbs 0.46–0.57 times SVL, hindlimbs 0.58–0.69 times SVL. Tail almost round in cross section, tapering toward the tip; 1.29–3.15 times SVL.

**Figure 4.**
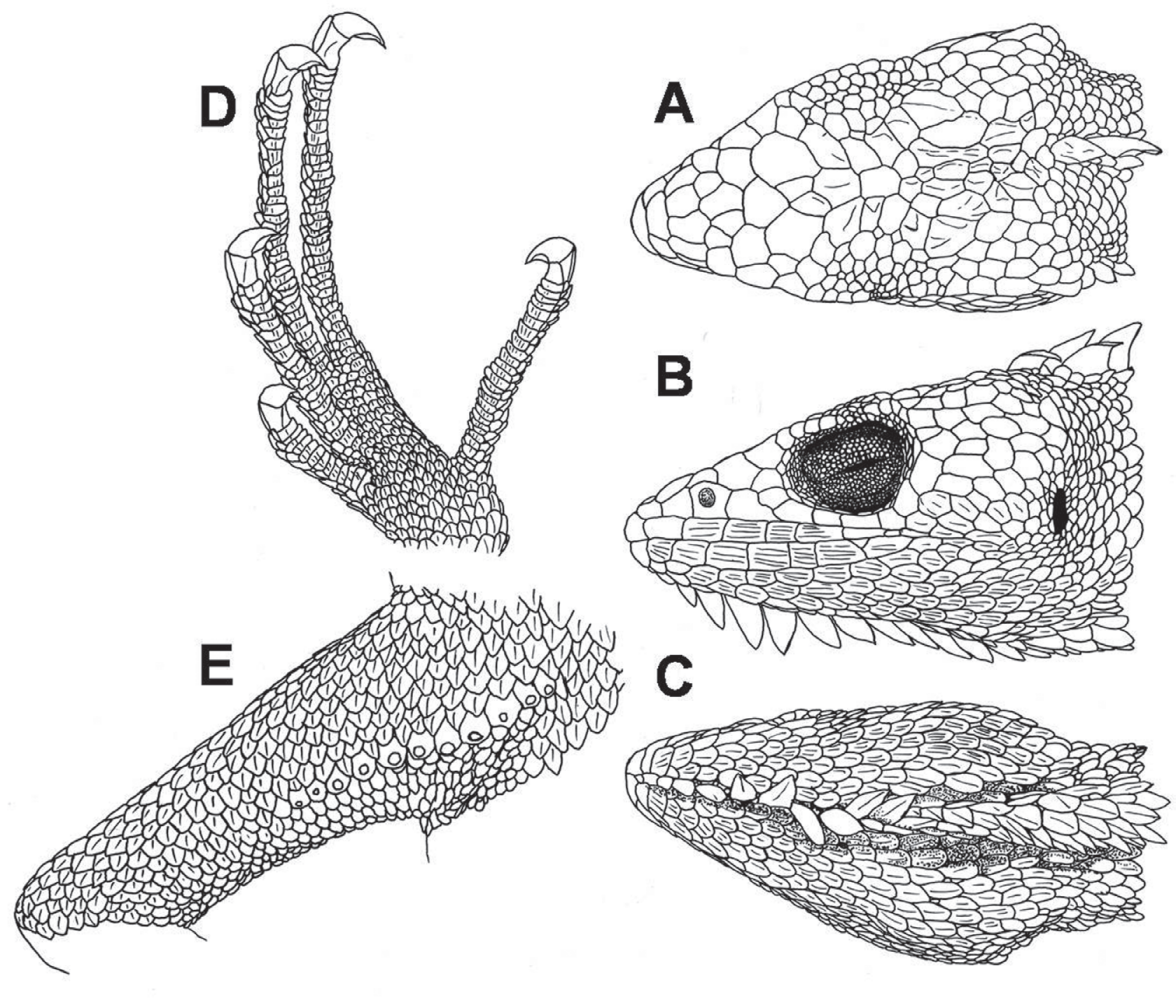
Male specimen of *Polychrus peruvianus* ZFMK 90821: dorsal **A**, lateral **B** and ventral **C** views of head; ventral aspect of left foot **D**, ventral view of right thigh with femoral pores **E**.

Rostral trapezoid, striated, about two times as wide as high. Most of the individuals (18/23) lack sutures on the posterior margin of the rostral, three specimens possess one very short median suture, one specimen exhibits a median suture that half divides the rostral and another specimen exhibits two short sutures on the posterior margin. Rostral bordered posteriorly by 2–4 scales, mostly 3 (17/23). Postrostral scales striated. Scales on snout heterogeneous in size, irregularly polygonal, juxtaposed, rugose or swollen; 1–4 scales, mostly 2 (14/23) across snout between second canthals. Two striated canthals between nasal and supraciliaries (3 in one specimen: ZFMK 90829). Supraorbital semicircles distinct, with 8–12 scales, separated medially by 1 scale (Fig. 4A). Scales on supraocular region distinctly smaller than those on snout, polygonal, juxtaposed, flat, smooth or slightly striated; irregularly arranged, except for a row of smaller scales adjacent to the supraciliaries. Supraciliaries 8–12 (n=23), juxtaposed, smooth; in a continuous series with canthals. Scales on parietal region, irregular polygonal, juxtaposed, flat, smooth or slightly striated, slightly smaller than those on snout. Scales on interparietal region polygonal, juxtaposed, rugose or swollen, almost the same size as those on the parietal region. Parietal eye absent. Loreal region has one striated scale. Nostril directed laterally, in the centre of a single nasal or slightly anterior to the center. Nasal scale has polygonal margins and is in broad contact with second supralabial. 3–6 internasals. Eye diameter 0.25–0.31 (n=23) times as long as head length. Eyelids partially fused together, covered by granules of almost same size throughout the eyelids. A continuous series of 1–3 preoculars, 2–4 suboculars, which are in direct contact with supralabials, and 3–5 postoculars. Supralabials 5–10, strongly striated with 2–5 keels; followed to commissure of mouth by 2–4 slightly smaller scales. Temporal region has polygonal or rounded, juxtaposed, flat, and smooth or slightly striated scales, nearly the same size as those of parietal region; delimited dorsally by a single row of 3–5 (n=23) enlarged supratemporal scales. Ear opening, vertically oval, with smooth margin; tympanum superficial (Fig. 4B).

Mental striated, two to 2.5 times as wide as high, posteriorly notched, followed by a median sulcus that almost or at least divides the mental half. Postmentals 3–4 (n=23), striated, lateral ones larger than median scale. Infralabials 5–10, strongly striated with 3–8 keels; followed to commissure by 2–4 distinctly smaller scales. Lateral scales on chin and gular flap oval, in posterior part more or less drawn-out, imbricate, flat and strongly striated with 1–8 keels. A row of 8–14 (n=23) raised, lobe-shaped, striated scales forming a mid-chin crest and merging into a gular flap that reaches the posterior level of the forelimbs (Fig. 4C). 28–38 (n=23) gular scales in transverse line between the two tympani. In posterior part of gular fan, most of the scales are separated from each other by a narrow stripe of extensible skin covered with granules.

Scales on nape anteriorly relatively small, almost rounded, juxtaposed and convex; posteriorly grade into dorsals and merge ventrally with gulars. Middorsal crest present; in adult males it is composed of 20–28 lobe-shaped scales, reaching from behind the occiput to the level of the hindlimbs, in females or juvenile males it is composed of 7–19 lobe-shaped scales, present only on anterior part of the dorsum. Lateral dorsals are oval or slightly lanceolate and are almost the same size throughout body, imbricate, flat; unicarinate in paravertebral region; number of keels augments in direction of ventral body part.

56–70 scales in a paravertebral line between occiput and posterior margin of hindlimbs. Ventrals imbricate, distinctly more overlapping and slightly smaller than dorsals, strongly lanceolate, uni- to multicarinate; in thorax region slightly smaller, in abdominal region arranged in oblique and transverse rows. A gradual transition between dorsal, lateral and ventral scales. Scales around midbody 52–74 (n=47). Preanal pores absent. Femoral pores 6–13 (n=47) (Fig. 4E).

Tail with imbricate, rhomboid, flat, sharply keeled scales, slightly larger than dorsals; in longitudinal and oblique rows, keeles aligned longitudinally. Original tail ending more or less pointed.

Scales on forelimbs slightly smaller than dorsals, imbricate and more or less lanceolate, uni- to tricarinate. Scales on hindlimbs slightly smaller than dorsals, imbricate and more or less lanceolate, unicarinate on dorsal surface and uni- to tricarinate on ventral surface. Subdigital lamellae of fingers and toes single, short, multicarinate, 25–33 (n=47) under fourth finger, 32–43 (n=47) under fourth toe (Fig. 4D).

**Figure 5.**
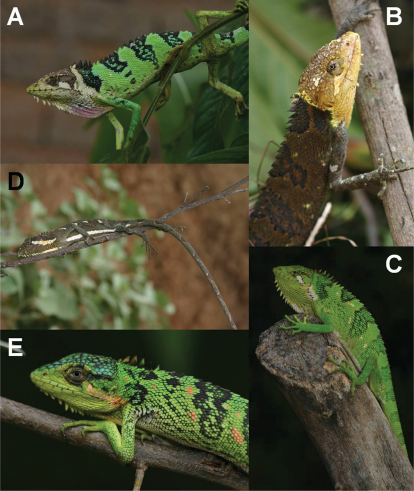
*Polychrus peruvianus* from Cajamarca, Peru: male with normal colouration **A** CORBIDI 1852, in stress colouration **B** CORBIDI 1846, female with normal colouration **C** ZFMK 88712, in stress colouration **D** ZFMK 90819, a very colourful juvenile female **E** CORBIDI 5725.

In life, when unstressed, the dorsal ground colouration of males (Fig. 5A) and females (Fig. 5C), is lime green on body, limbs and tail. Back and tail with dark blotches that are at least as broad as the green interspaces, with the first blotch beginning directly behind the head in females, or adjacent to a small white nuchal crossline in most males. Most specimens possess 5 of such saddle blotches on the dorsum, which are broadest in the vertebral region and decrease in width on the flanks. Blotches are more distinct in males, and are rarely found, or even absent, in females, and normally intermixed with scales of green ground colour. Additionally, some specimens possess white or pinkish and/or turquoise scales or small blotches on the lateral body parts (Fig. 5E). Head in females dorsally, laterally and ventrally lime green; in males dorsally and laterally brownish or orange brown and in some individuals spotted with white, ventrally lighter brown or yellowish, sometimes almost whitish. Scales of gular crest are white in most specimens of both sexes and extensible skin of exposed gular sac is orange, yellowish or pinkish (Fig. 5A). Females mostly with an oblique white line on both sides from behind the eye to the insertion of the forelimbs and with a straight line, about 3 to 4 scales in width, laterally between the axilla and the insertion of the hindlimbs. Venter of both sexes, lime green without special markings.

Under stress, colouration of body, limbs and tail changes into a dark brown in both sexes (Fig. 5B, D), in which case the dark saddle blotches become less evident. If the animal possesses white markings, these become even more prominent. Head colouration of females (Fig. 5D) changes into dark brown, but remains as in the unstressed mood in males (Fig. 5B).

In preservative, dorsal pattern remains similar to the pattern in life but colouration changes into bluish or brownish. Heads of males are dorsally and laterally brownish, and ventrally cream colour or whitish. Venter of both sexes pale blue, green or brown.

#### Distribution and natural history. 

In Peru, this species is distributed in the regions of Amazonas, Cajamarca, and Piura in the drainage basins of Río Huancabamba, Río Utcubamba and Río Marañón (Schlüter 2010, Noble 1924, Gorman et al. 1969, Peters and Donoso-Barros 1970, Carrillo and Icochea 1995). Yánez-Muñoz et al. (2006) collected a male specimen from Pucabamba (04°57'01"S, 79°10'30"W, 1400 m a.s.l.), Province of Zamora-Chinchipe, and hence provided the first country record from Ecuador. *Polychrus peruvianus* inhabits the equatorial dry forest eco-region fide Brack (1986), but is also occasionally found in humid forests, at elevations of 600 to 1750 m a.s.l. (Duellman 1979; Gorman et al. 1969; Noble 1924). We found the species at an elevation of 400 to 1330 m above sea level. Besides the few specimens we collected for preservation, we found many more animals of the same species in each sampled area and noted additional observations we could make. All lizards were exclusively found on trees or shrubs (preferred plant species: *Acacia macracantha*, *Acacia riparia*, *Hura crepitans*, *Mutingia calabura*, *Sapindus riparium*, *Schinus molle*, *Solanum riparium*) alongside roads, paths, or small streams in heights between 1.5 m and 7 m above the ground. Hence the species can be considered as being highly arboreal. Only some specimens were found during the day (investigation hours: 9.30 a.m. to 4 p.m.) as they are perfectly camouflaged in the vegetation and difficult to detect between the green leaves. Daytime temperatures, when animals were found, were between 28.7°C and 35.9°C and humidity was between 41% and 63%. Most specimens were discovered after nightfall (investigation hours: 7 p.m. to 2 a.m.), when they were sleeping on branches and their bellies were shining brightly in the light of the headlamps. Nighttime temperatures were between 20.8°C and 28.3°C and humidity was between 53% and 75%. In Pucará, one individual could be observed at around 10 a.m., while it was eating little fruits of the tree *Trema micrantha*. Several times we found two, sometimes even three, specimens sleeping on the same tree. In Pucará, the species seemed to be very abundant and in one night we counted 24 adult and 3 juvenile specimens on 22 trees along a two kilometer long path section. One male and one female were found about only 0.5 m away from each other. This represents the encounter with the lowermost distance between two individuals. Other individuals were found with a distance of at least 1–2 m to the next conspecific, irrespective of sex. Although it seems that members of this species have small activity ranges, they live solitarily. Adult males exhibit a pronounced territorial behaviour and do not tolerate other males close to their branches. Under artificial conditions, a male being confronted with another male or even with its own mirror image, opened its mouth widely and extended its gular flap. Efforts to keep two males together in a cage of 3 × 2 m floor space and 2 m in height started with a non-ritualized damaging fight which lasted for around 10 minutes. After the fight the bigger male persecuted the other male in the cage and two days later the smaller male was found dead.

When discovered in a tree, the animals first react similarly as a chameleon: they compress their body laterally and try with very slow movements to take cover behind a stick or branch. Once grabbed, they expand their gular fan, open their mouth widely and try to bite the captor while they try, simultaneously, to free their bodies with strong twisting and turning movements. Similar observations were also made by Gorman et al. (1969) for *Polychrus peruvianus* and by Vanzolini (1983) for the genus *Polychrus* in general. In addition, we could observe a change in colouration in most captured animals to the above described stress colouration.

One gravid female (ZFMK 90822) was found in April 2009 at 10.35 p.m. sleeping in a tree at about 2.5 m above the ground, with an air temperature of 24.9°C and a humidity of 73%. It contained 5 oval eggs, 3 in the left and 2 in the right oviduct. In average these eggs had a length of 27.5 mm and a width of 16.2 mm. In December 2009, we collected 4 gravid females (ZFMK 90824, 90827, 90829, 90830) in different stages of gestation between 8–10.30 p.m. sleeping on trees in 2–5.5 m above the ground. Air temperature was between 25.5°C–28°C and humidity was between 55–75%. ZFMK 90824 contained 10 almost spherical eggs with a diameter of 12 mm, of which 7 were positioned in the left and 3 in the right ovary. ZFMK 90827 contained 7 almost spherical eggs with a diameter of 6 mm of which 3 were positioned in the left and 4 in the right ovary. ZFMK 90829 contained 7 almost spherical eggs with a diameter of 8.9 mm of which 4 were positioned in the left and 3 in the right ovary. ZFMK 90830 contained 4 almost spherical eggs with a diameter of 9.4 mm, 2 were positioned in each of the ovaries.

### Polychrus gutturosus

Berthold, 1845

http://species-id.net/wiki/polychrus_gutturosus

Polychrus gutturosus  Berthold, Nachrichten von der Georg-Augusts Universität und der Königlichen Gesellschaft der Wissenschaften zu Göttingen, 3: 38. — Terra typica: Popayán, western Colombia. – 1845
Polychrus gutturosus  – Berthold, Nachrichten von der Georg-Augusts Universität und der Königlichen Gesellschaft der Wissenschaften zu Göttingen, 8-10: 11. – 1846
Polychrus (Chaunolaemus) multicarinatus  Peters, Monatsberichte der königlich Akademie der Wissenschaften zu Berlin 1869 (11): 768. – Terra typica: Costa Rica.  – 1869
Polychrus gutturosus  – Boulenger, Catalogue of the lizards in the British Museum, 2: 100. – 1885
Polychrus spurrelli  Boulenger, Proceedings of the Zoological Society of London, 1914: 814. – Terra typica: near Peña Lisa, Condoto, Colombia. – 1914
Polychrus gutturosus  – Burt and Burt, Transactions of the Academy of Science of St. Louis, 28: 40. – 1933
Polychrus gutturosus gutturosus  – Parker, Proceedings of the Zoological Society of London, 105 (3): 516. – 1935
Polychrus gutturosus spurrelli  – Parker, Proceedings of the Zoological S﻿﻿ociety o﻿﻿f London, 105 (3): 516. – 1935
Polychrus gutturosus  – Etheridge, Herpetologica, 21: 167. – 1965
Polychrus gutturosus  – Peters and Donoso-Barros, Smithsonian﻿﻿ I﻿﻿﻿﻿nstitution Press, Washington D.C. & London: 233. – 1970
Polychrus gutturosus spurrelli  – Peters and Dono﻿﻿so-Barros, Smithsonian Institution Press, Washington D.C. & London: 234. – 1970
Polychroides gutturosus  – Peters and Donoso-Barros, ﻿﻿Smithsonian Institution Press, Washington D.C. & London: 233. – 1986
Polychrus gutturosus spurrelli  – Peters and Donoso-Barros, Smithsonian Institution Press, Washington D.C. & London: 234. – 1986
Polychrus gutturosus  – Roberts, Herpetological Review, 28 (4): 184. – 1997
Polychrus gutturosus  – Köhler, Herpeton Verlag, Offenbach: 83. – 2000
Polychrus spurrellii  – Torres-Carvajal, Smithsonian Herpetological Information Service, 131: 21. – 2001
Polychrus gutturosus  – Savage, University of Chicago Press, 2﻿﻿nd edition: 445. – 2002
Polychrus gutturosus  – Köhler, Herpeton Verlag, Offenbach: 13﻿﻿7. – 2003
Polychrus spurrelli  – Yánez-Muñoz et al., Check List, 2 (2): 63. – 2006

#### Diagnosis

**Figure 6.**
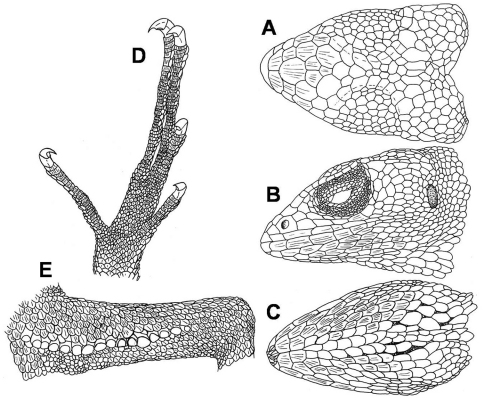
Male specimen of *Polychrus gutturosus* (SMF 83024): dorsal **A** lateral **B** and ventral **C** views of head; ventral aspect of right foot **D** ventral view of left thigh with femoral pores **E**.

 (Tab. 3). (1) A *Polychrus* with a maximum known SVL of 170 mm; (2) dorsal and gular crest absent; (3) 63 to 82 scales around midbody; (4) 75 to 105 scales in middorsal row from behind the occipital scales to the level of the posterior edge of the thigh; (5) femoral pores 9 to 21 on one side (Fig. 6E); (6) lamellae on finger IV 25–36; (7) lamellae on toe IV 35–45 (Fig. 6D); (8) tail 2.36–3.55 times longer than SVL; (9) dorsal scales smooth or with 1–3 keels; (10) ventral scales with 1–5 keels; (11) gular scales oval, mostly striated, much larger than ventrals, those on gular fan widely separated by granular skin (Fig. 6C); (12) a sexual dichromatism present.

**Table 3.** Summary of morphometric and pholidosis characters of *Polychrus gutturosus*

Sex	All# (n=27)	Males (n=10)	Females (n=15)
Axilla-groin length/SVL	0.45–0.61<br/> (0.53 ± 0.03)	0.45–0.55<br/> (0.53 ± 0.03)	0.45–0.61<br/> (0.53 ± 0.04)
Head length/SVL	0.16–0.25<br/> (0.22 ± 0.02)	0.18–0.25<br/> (0.22 ± 0.02)	0.16–0.24<br/> (0.22 ± 0.02)
Head length/Head width	1.10–1.6<br/> (1.42 ± 0.15)	0.93–1.56<br/> (1.38 ± 0.20)	1.10–1.6<br/> (1.44 ± 0.13)
Head width/Head height	0.9–1.34<br/> (1.11 ± 0.13)	0.97–1.34<br/> (1.07 ± 0.13)	0.9–1.29<br/> (1.14 ± 0.14)
Tail length/SVL	2.36–3.55<br/> (3.10 ± 0.28)	3.08–3.55<br/> (3.30 ± 0.16)	2.36–3.55<br/> (2.99 ± 0.28)
Scales around midbody	63–82<br/> (73.0 ± 5.4)	63–75<br/> (68.4 ± 3.27)	66–82<br/> (76.33 ± 4.42)
Vertebral scales	75–105<br/> (89.15 ± 7.15)	75–93<br/> (84.6 ± 5.56)	84–105<br/> (93.13 ± 5.97)
Gular scales	22–33<br/> (28.59 ± 2.36)	22–30<br/> (26.9 ± 2.28)	27–33<br/> (29.8 ± 1.78)
Diameter eye/head length	0.27–0.49<br/> (0.33 ± 0.05)	0.30–0.49<br/> (0.35 ± 0.05)	0.27–0.41<br/> (0.31 ± 0.03)
Subdigitals finger IV	25–36<br/> (31.52 ± 2.79)	30–36<br/> (33.0 ± 1.94)	25–36<br/> (30.87 ± 2.8)
Subdigitals toe IV	35–45<br/> (40.81 ± 2.43)	38–44<br/> (41.2 ± 2.15)	37–45<br/> (41.07 ± 2.28)
Forelimbs/SVL	0.37–0.54<br/> (0.47 ± 0.04)	0.37–0.54<br/> (0.49 ± 0.04)	0.38–0.53<br/> (0.46 ± 0.04)
Hindlimbs/SVL	0.46– 0.73<br/> (0.59 ± 0.06)	0.56– 0.73<br/> (0.63 ± 0.06)	0.46– 0.63<br/> (0.57 ± 0.05)
Femoral pores (left)	9–21<br/> (14.76 ± 3.15)	14–19<br/> (15.7 ± 1.49)	9–21<br/> (13.93 ± 3.87)

^#^ 10 males, 15 females, 2 juveniles

#### Description

**.** For detailed descriptions of shape, structure and arrangement of the scales see Taylor (1956) and Savage (2002).Our examined female specimens (n=15) had a maximum SVL of 152 mm, a maximum tail length of 539 mm, a maximum total length of 691 mm, a maximum head length of 33.3 mm and a maximum head width of 26.4 mm. The male specimens (n=10) had a maximum SVL of 122 mm, a maximum tail length of 429 mm, a maximum total length of 549.8 mm, a maximum head length of 28.1 mm and a maximum head width of 22.2 mm. Rostral bordered posteriorly by normally 4 striated scales (3 in one specimen: ZFMK 40832; 5 in another specimen: MHNG 2531.062). Scales on snout heterogeneous in size, irregularly polygonal, juxtaposed, rugose and striated; 1–6 scales across snout between second canthals. 2–3 striated canthals between nasal and supraciliaries. Supraorbital semicircle distinct (Fig. 6A), with 7–13 scales, separated medially by normally one scale (0 in two specimens: BM 1901.3.29.19, MHNG 2531.062; 2 in another specimen: ZFMK 19047). Supraciliaries 7–11, juxtaposed, striated; in a continuous series with canthals. Supraocularies 12–18. Internasals 3–5.Supralabials 4–8, strongly striated with 2–6 keels; followed to commissure of mouth by 2–4 slightly smaller scales. Infralabials 4–6, strongly striated with 3–8 keels; followed to commissure by 1–4 distinctly smaller scales (Fig. 6B). Mental approximately half divided by a median groove in 17 specimens, almost divided in 5 specimens, medially divided in one specimen and divided into numerous small scales in one specimen. Postmentals striated (Fig. 6C), normally 2 (5 in one specimen: ZFMK 25729). Supratemporals 4–5; scales in temporal region striated.

Paravertebral scales mostly keeled, only some are smooth; lateral body scales smooth or with 1–3 keels, fore- and hindlimbs dorsally with one or more keels, ventrally multicarinate. Ventral body scales with 1–5 keels.

Other morphological characters of the 27 examined individuals are summarized in Table 3.

**Figure 7.**
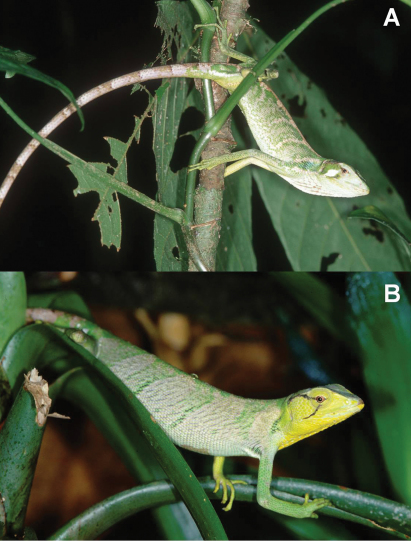
*Polychrus gutturosus* from near Río San Juan, Nicaragua (photographs by G. Köhler): male  **A** SMF 83024 and female **B** SMF 83422.

Descriptions of the colouration in life (Fig. 7A, B) are given by Breder (1946), Köhler (2003b), Ortleb and Heatwole (1965) and Savage (2002) and a description of the colour in preservative is provided by Taylor (1956).

#### Distribution and natural history.

 From northwestern Honduras and western Costa Rica to northwestern Ecuador (Köhler 2003a, Savage 2002) from sea level to 1300 m elevation (Castro-Herrera and Vargas-Salinas 2008). According to Duellman (1979), the species occurs on the Pacific slopes of the Cordillera Occidental in Colombia and Ecuador, the northern parts of the Colombian cordilleras and in the high lands in lower Central America. According to Peters (1967)and(Peters and Donoso-Barros (1970, 1986), *Polychrus gutturosus gutturosus* is distributed from the higher western Andean slopes of Ecuador and Colombia and northward to Costa Rica and Nicaragua; whereas *Polychrus gutturosus spurrelli* occurs in lowland rain forests of northwestern Ecuador and Colombia. According to Savage (2002), the species occurs in undisturbed lowland moist and wet forests and marginally along stream courses which lead into the adjacent Premontane Moist Forest.

Despite its restriction to humid forests, it is strictly diurnal and arboreal and is rarely seen (Savage 2002). A female specimen from Turrialba, Cartago Province, Costa Rica examined by Taylor (1956) contained 4 ovarian eggs in the right and 5 in the left ovary, respectively. Eggs were nearly spherical and measured 12 mm in diameter. Roberts (1997) observed a pair of *Polychrus gutturosus* copulating in a tree 2 m above the ground at La Selva Biological Station, Heredia Province, Costa Rica on 9 May and further reports of a gravid female, that was found in a *Heliocarpus* sp. tree next to Puerto Viejo river at La Selva on 24 July. According to Savage (2002), juveniles have a SVL of 53.5–57 mm when hatching. We examined 2 juveniles with a SVL of 87 mm (ZFMK 31444) and only 44 mm (QCAZ 06749), respectively. Two specimens which were not examined any further had a SVL of 39 mm (BM 94.5.29.5) and 57 mm (BM 1901.3.29.84), respectively. Based on the so far reported cases of copulating animals and gravid females, Savage (2002) suggested a rainy season productive period (May to December). He stated that eggs are apparently laid in the leaf litter on the ground. Köhler (2003B) kept a couple of *Polychrus gutturosus* from near Rio San Juan, Nicaragua, in a terrarium. On 26 October the female laid 5 eggs, which decayed and could not be incubated successfully. We examined a gravid female (ZFMK 40830) from Comatré, Limón, Costa Rica, which was collected in October 1983. It contained a total of 6 oval eggs of which 3 were positioned in each of the oviducts. On the average, these eggs had a length of 21 mm and a width of 15 mm.

## Discussion

*Polychrus peruvianus* is the only representative in the genus with a prominent middorsal and gular crest. Due to this character it was originally described as belonging to a new genus *Polychroides* (Noble 1924). Burt and Burt (1933) followed this nomenclature, whereas Parker (1935) and Roberts (1997) accepted only 5 species of *Polychrus* and thus consider *peruvianus* as not belonging to this genus. In contrast, Williams (1988) and Savage (2002) recognized 7 species of *Polychrus*, signifying that they considered *peruvianus* as belonging to this genus. Osteological (Etheridge 1965, Etheridge and De Queiroz 1988) and cytological data (Gorman et al. 1969) show a very close relationship to the genus *Polychrus*, andlead the authors to the assumption that *peruvianus* belongs to this genus. Due to phylogenetic examination of morphological data, Frost et al. (2001) placed *peruvianus* in the genus *Polychrus*. Yánez-Muñoz et al. (2006), who provided the first country record of the species for Ecuador, also considered the species as belonging to the genus *Polychrus*. Analysis of molecular data is still lacking to definitely determine the position of this species.

*Polychrus gutturosus* is the only species in the genus assumed to be composed of two subspecies (*Polychrus gutturosus gutturosus* and *Polychrus gutturosus spurrelli*). However, disagreement still exists on the status of the latter, which was described by Boulenger (1914) as a distinct species *Polychrus spurrelli* and later placed as a subspecies of *Polychrus gutturosus* (Parker 1935). According to Parker's identification key, the pectoral scales of *Polychrus gutturosus gutturosus* are multicarinate whereas those of *Polychrus gutturosus spurrelli* are smooth. Peters (1967) and (Peters and Donoso-Barros (1970, 1986) also consider *spurrelli* as a subspecies of *Polychrus gutturosus*. According to the key provided by them, the canthus rostralis is somewhat rounded and the scales on the pectoral region are smooth, or only very weakly keeled in *Polychrus gutturosus spurrelli*, whereas in *Polychrus gutturosus gutturosus* the canthus rostralis is distinctly angular and the scales on the pectoral region are strongly keeled, usually unicarinate but may be bi- or tricarinate. According to Frost et al. (2001), Pough et al. (2004), and Avila-Pires (1995) the genus *Polychrus* contains 6 species and according to Roberts (1997), it contains only 5 species. Hence these authors do not accept *spurrelli* as being a distinct species. In a species list of Colombian lizards provided by Ayala (1986), the only *Polychrus* species mentioned to occur in the country are *Polychrus gutturosus* and *Polychrus marmoratus*, equally revealing that the author did not accept *spurrelli* as a valid species. In contrast, Williams (1988) and Savage (2002) accept 7 species in the genus *Polychrus*, which signifies that they considered it as a distinct species. Torres-Carvajal (2001) and Yánez-Muñoz et al. (2006) also considered *Polychrus spurrelli* as a valid species.

As explained in very detail by Myers and Böhme (1996), it is not sure whether the type locality provided by Boulenger (1914) for *Polychrus gutturosus* is really the highland city Popayán (1760 m a.s.l.), as referenced to by several authors (e.g. Barbour 1934, Peters and Orejas-Miranda 1970, Myers 1974), but rather a colonial province named Popayán which seems to have existed until 1820 and which once included nearly all of what is now western Colombia. Thus, the chance is quite high that the type specimen of P. gutturosus was originally collected at some other place in western Colombia and probably at lower elevation. Hence the original location of *Polychrus gutturosus* within the old province of Popayán cannot be determined, and the existence of a geographic isolation of *Polychrus gutturosus* and *Polychrus spurrelli* is not proven. The assumption that both taxa represent different subspecies of *Polychrus gutturosus* (i.e. by definition allopatric forms) is not supported. To shed light on the taxonomic status of *spurrelli*, we revised the two syntypes (BM 1946.8.8.33–34) on which Boulenger (1914) based his species description and two further specimens (BM 1916.4.25.2–3) in the British Museum of Natural History which were also collected by Dr. H.G.F. Spurrell in Colombia (Andagoya, Chocó) and were also designated as *Polychrus spurrelli*. The two syntypes represent subadult females and the two other specimens represent adult males. We could not find any difference in either morphometric or pholidosis characters (Tab. 4) or in colouration between these four *Polychrus* and the 27 specimens of *Polychrus gutturosus* formerly examined for this study. The shape of the canthus rostralis is more rounded in some specimens, whereas it is more angular in others. We found the scales in the pectoral region of the four *spurrelli* to be smooth, or slightly uni- or tricarinate in one specimen (BM 1946.8.8.34), uni- or tricarinate in two specimens (BM 1946.8.8.33, BM 1916.4.25.2) and multicarinate with 2–5 keels in one specimen (BM 1916.4.25.3). Again, there was no difference to the specimens of *Polychrus gutturosus* studied by us, which exhibited pectoral scales with 1–5 keels.

**Table 4.** Summary of morphometric and pholidosis characters of *Polychrus spurrelli*

Sex	All (n=4)	Males (n=2)	Females (n=2)
Axilla-groin length/SVL	0.5–0.55<br/> (0.52 ± 0.02)	0.52–0.54<br/> (0.53 ± 0.01)	0.5–0.55<br/> (0.52 ± 0.01)
Head length/SVL	0.19–0.24<br/> (0.21 ± 0.03)	0.19<br/> (0.91 ± 0.0)	0.24<br/> (0.24 ± 0.03)
Head length/Head width	1.16–1.84<br/> (1.49 ± 0.28)	1.16–1.84<br/> (1.50 ± 0.48)	1.41–1.55<br/> (1.48 ± 0.17)
Head width/Head height	0.9–1.12<br/> (1.00 ± 0.08)	0.93–0.95<br/> (0.94 ± 0.01)	1.01–1.12<br/> (1.07 ± 0.13)
Tail length/SVL	2.97–3.20<br/> (3.07 ± 0.12)	2.97–3.20<br/> (3.09 ± 0.16)	2.97–3.14<br/> (3.06 ± 0.0)
Scales around midbody	64–82<br/> (67.5 ± 4.73)	64–74<br/> (66.0 ± 2.83)	64–68<br/> (69.0 ± 2.83)
Vertebral scales	85–94<br/> (87.75 ± 4.72)	85–87<br/> (86.0 ± 1.41)	85–94<br/> (89.5 ± 1.41)
Gular scales	24–29<br/> (27.25 ± 2.36)	27–29<br/> (28.0 ± 1.41)	24–29<br/> (26.5 ± 3.54)
Diameter eye/head length	0.35–0.42<br/> (0.38 ± 0.03)	0.40–0.42<br/> (0.41 ± 0.01)	0.35<br/> (0.35 ± 0.04)
Subdigitals finger IV	27–31<br/> (29.0 ± 1.63)	29–31<br/> (30.0 ± 1.41)	27–29<br/> (28.0 ± 1.41)
Subdigitals toe IV	36–38<br/> (37.25 ± 0.96)	36–38<br/> (37.0 ± 1.41)	37–38<br/> (37.5 ± 0.71)
Forelimbs/SVL	0.43–0.51<br/> (0.47 ± 0.04)	0.45–0.51<br/> (0.48 ± 0.04)	0.43–0.50<br/> (0.46 ± 0.06)
Hindlimbs/SVL	0.54– 0.64<br/> (0.60 ± 0.04)	0.54– 0.60<br/> (0.57 ± 0.04)	0.63– 0.64<br/> (0.64 ± 0.02)
Femoral pores (left)	12–15<br/> (13.75 ± 1.26)	14–15<br/> (14.5 ± 0.71)	12–14<br/> (13.0 ± 2.12)

Based on our observations, there is no evidence to support the recognition of *Polychrus spurrelli* as a distinct species; thus, we synonymize it here with *Polychrus gutturosus*. Genetic examination could further help to better determine the status of *spurrelli*.

Our field work resulted in the discovery of yet another undescribed species of *Polychrus* from northern Peru. We will provide a comprehensive description of this new species in a further publication.

## Supplementary Material

XML Treatment for Polychrus jacquelinae

XML Treatment for Polychrus peruvianus

XML Treatment for Polychrus gutturosus

## Appendix I: specimens examined

*Polychrus peruvianus*–PERU: *Cajamarca*: Jaén: Santa Rosa (05°26'S, 078°33'W, 1250–1300 m a.s.l.), ZFMK 88710, CORBIDI 5731, CORBIDI 5732; Bellavista (05°39'49.8"S, 78°40'13.9"W, 411 m a.s.l.), CORBIDI 1863, CORBIDI 1857–8, ZFMK 88707; (05°38'06.6"S, 078°39'36.2"W, 405 m a.s.l.), CORBIDI 5728, ZFMK 90819, CORBIDI 5727, ZFMK 90818; (05°34'35.7"S, 078°38'10.8"W, 700 m a.s.l.), ZFMK 90820; Gota de Agua (05°41'S, 078°46'W), ZFMK 88708; Perico (05°21'16.5"S, 078°47'30.6"W, 443 m a.s.l.), CORBIDI 1933, ZFMK 88709; Perico (05°21'S, 078°47'W, 460–720 m a.s.l.), CORBIDI 5730, ZFMK 90822, CORBIDI 5729, ZFMK 90821; Pucará (06°02'S, 079°07'W, 900–930 m a.s.l.), ZFMK 88711, CORBIDI 1846, CORBIDI 5726, ZFMK 90817, CORBIDI 5725, CORBIDI 5724; *Amazonas*: Bagua: Bagua Grande (05°47'33.3"S, 078°23'04.9"W, 570 m a.s.l.), ZFMK 88712, CORBIDI 1852, ZFMK 88713; Utcubamba: Zapatalgo (06°04'S, 078°29'W, 900–130 m a.s.l.), CORBIDI 5733, ZFMK 90824, CORBIDI 5734, ZFMK 90823, CORBIDI 5735, ZFMK 90825; Puerto Malleta: (06°03'S, 078°36'W, 480–510 m a.s.l.), ZFMK 90824, 90826–7, CORBIDI 5736–8, ZFMK 90828; (06°04'S, 078°36W, 535 m), ZFMK 90829, CORBIDI 5739; Cumba (05°56'S, 078°39'W, 450–500 m a.s.l.), CORBIDI 5741, ZFMK 90830, CORBIDI 5740, ZFMK 90831–2, ZFMK 90833.

*Polychrus gutturosus*–COLOMBIA: BM 1923.10.12.16; *Chocó*: Condoto, ca.100 m: BM 1913.11.12.18–19; *Popayan*: ZFMK 21341 (holotype); COSTA RICA: ZFMK 19047, ZFMK 25729, ZFMK 45355 (from animal trade, place and date not further specified); Pozo Azul: BM 1907.6.28.2; *Turrialba*, 900 m: ZFMK 31444 (from animal trade in 1980); *Limón*: Comatré: ZFMK 40830–31; *Punta Arenas*: Palmar: ZFMK 40832, ZFMK 40833; NICARAGUA: Rio San Juan: near Rio San Juan, SMF 83024 (from animal trade on 21 May 2001, place not further specified); PANAMA: BM 94.6.29.10; ECUADOR: St. Javier (NW Ecuador): BM 1901.3.29.19–20; Paramba: BM 98.4.28.33; *Esmeraldas*: Comunidad Selva Alegre, Rio Santiago: QCAZ 3490, QCAZ 6934, QCAZ 8817, QCAZ 9788; *Pichincha*: La Union del Toachi: QCAZ 6749; Santo Domingo de los Colorados: MHNG 2437.029, MHNG 1117.036; *Cotopaxi*: San Francisco de Las Pampas: MHNG 2437.028; *Imbabura*: Lita: MHNG 2531.062.

*Polychrus spurrelli*–COLOMBIA: *Chocó*: Condoto: Peña Lisa: BM 1946.8.8.33–34 (syntypes); Andagoya: BM 1916.4.25.2–3.
